# Intergenerational transmission of appetite: Associations between mother-child dyads in a Mexican population

**DOI:** 10.1371/journal.pone.0264493

**Published:** 2022-03-15

**Authors:** Claudia Hunot-Alexander, Carmen Patricia Curiel-Curiel, Enrique Romero-Velarde, Edgar Manuel Vásquez-Garibay, Alethia Mariscal-Rizo, Erika Casillas-Toral, Andrea Dominica Smith, Clare Heidi Llewellyn

**Affiliations:** 1 Instituto de Nutrición Humana, Centro Universitario de Ciencias de la Salud, Universidad de Guadalajara, Guadalajara, México; 2 Centro Universitario de Tonala, Universidad de Guadalajara, Tonala, Jalisco, México; 3 Behavioural Epidemiology and Interventions in Young People Group, MRC Epidemiology Unit, University of Cambridge, Cambridge, United Kingdom; 4 Department of Behavioural Science and Health, University College London, London, United Kingdom; Mugla Sitki Kocman Universitesi, TURKEY

## Abstract

The Child Eating Behaviour Questionnaire (CEBQ) and the Adult Eating Behaviour Questionnaire (AEBQ) measure ‘food approach’ [Food responsiveness (FR); Emotional overeating (EOE); Enjoyment of food (EF); Desire to Drink] and ‘food avoidant’ [Satiety responsiveness (SR); Emotional undereating (EUE); Food fussiness (FF); Slowness in eating (SE)] appetitive traits (ATs) in children and adults, respectively. ‘Food approach’ traits predispose to overweight while ‘food avoidance’ traits provide protection, but little is known about the relationships between parents’ and their offspring’s ATs. The aim was to examine the associations between maternal and child appetitive traits, using the AEBQ-Esp and CEBQ-Mex adapted for use in Mexican populations. Sociodemographic data, weights and heights of mothers and their children (aged 3–13 years), who were recruited from a teaching hospital in Guadalajara, Mexico, were measured. Mothers completed both the AEBQ-Esp and the CEBQ-Mex. The CEBQ-Mex was developed, and its reliability was tested using Cronbach’s alpha and Omega, and Confirmatory Factor Analysis (CFA) was used to assess its validity. Pearson’s correlation coefficients were used to assess associations between mothers’ and children’s Ats. The sample included 842 mother-child dyads (mother’s mean age = 34.8±SD6.9 years, BMI 29.7±6.1 kg/m^2^; children’s mean age = 8.5 ±SD2.5 years, BMIz 1.5±1.6). Internal reliability was moderate to high [Cronbach alpha = .68-.86; Omega = .71-.87] for the CEBQ-Mex and validity was confirmed for an 8-factor model through CFA [RMSEA = 0.065; CFI = 0.840, NFI = 0.805; IFI = 0.842; and χ^2^(df = 532) = 2939.51, p < 0.001]. All but one of the children’s appetitive traits showed small to moderate, significant correlations with their mother’s counterpart [FR (*r =* .22; p<001); EOE (*r =* .30; p < .001); EF (*r =* .15; < .001); SR (*r =* .16; p < .001); EUE (*r =* .34; p < .001) and FF (*r =* .14; p < .001). Only SE was not significantly associated with maternal SE (*r =* .01; p>.05). ATs tend to run in families, signalling the intergenerational transmission of eating behaviours. These may be useful targets for family-wide interventions to support the development and maintenance of healthy eating behaviours in childhood.

## Introduction

Appetitive traits characterise an individual’s ability to self-regulate their food intake across multiple contexts and domains. They shape our drive to start or stop eating in response to food cues, the tendency to eat more or less in response to negative emotion, and our overall enjoyment of food.

Appetitive traits can be measured using a variety of methods. One approach is to assess eating behaviours in a laboratory setting, where a single meal time occasion (a ‘test meal’) is typically observed [1], but this is both expensive and time-intensive. Psychometric questionnaire measures offer a cheaper, quicker and less onerous alternative that allows data collection in larger samples. The Child Eating Behaviour Questionnaire (CEBQ) is a widely used psychometric instrument; the most commonly applied tool to study appetitive traits in children and one which was purposefully developed to measure these behaviours in relation to risk of developing overweight or obesity [2]. The CEBQ is parent-reported for children aged between 3 and 13 years. It consists of 35 items that measure four ‘food approach’ traits, which characterise a more avid appetite and a greater interest in food (Food Responsiveness, Emotional Over-Eating, Enjoyment of Food and Desire to Drink), as well as four ‘food avoidance’ traits that indicate a smaller appetite, better appetite regulation, and lower interest in food (Satiety Responsiveness, Emotional Under-Eating, Food Fussiness and Slowness in Eating). The CEBQ has subsequently been adapted for use in adult and adolescent populations. The same appetitive traits, with the exclusion of the Desire to Drink subscale, are included in the Adult Eating Behaviour Questionnaire (AEBQ), a self-report measure consisting of 30 items [3, 4].

A large evidence base using these questionnaires has established that variation in these traits has been linked to differences in weight across the entire BMI spectrum [3, 5–10]. In particular, food approach traits have been consistently positively associated with weight and are established risk factors for obesity, while food avoidance traits are consistently negatively associated with weight and protect against obesity, in both adults and children [3, 5–10]. Understanding how appetitive traits are shaped in early life is therefore a crucial part of obesity prevention.

In early life parents are the most dominant influences on their children’s eating behaviours [11], and they may influence them in multiple ways, including via modelling of eating behaviours themselves. A burgeoning research base has highlighted the importance of intergenerational transmission (i.e. concordance between parents’ and their children’s behaviour) of broad aspects of self-regulation (i.e. the ability to regulate cognition, emotion and behaviour), with parent-child associations observed across global measures. However, the relationship between parent and child self-regulation of appetite specifically has received much less attention, albeit studies have reported significant parent-child associations for disordered eating [12] and experimental measures of self-regulation of appetite [13]. Only one study to date has examined the associations between parent and child appetitive traits measured by the AEBQ and CEBQ, but the traits examined were limited. Miller and colleagues explored the relationship between maternal and child Food Responsiveness and Emotional Over-Eating in a sample of 478 Australian mother and child dyads. They found significant positive associations between the mothers’ and their child’s appetitive traits (r = .29 and r = .21, p < .001, respectively) [14]. However, only two of the seven appetitive traits were examined; we still know nothing about the parent-child relationships for the majority of the food approach and food avoidance traits measured by the AEBQ and the CEBQ.

A better understanding of the relationships between parents’ and their children’s appetitive traits could help provide information for clinicians to support parents to recognise certain appetitive traits in both themselves and their children. This may also help inform the development of behavioural components of clinical [15] and educational programmes aimed at the development of healthy eating habits in early childhood [16].

There were two primary aims of this study. Firstly, we report the factor structure and reliability of a Mexican-Spanish version of the CEBQ (CEBQ-Mex), developed for this study. Secondly, we use the AEBQ-Esp (Mexican-Spanish validated version [8]) and the CEBQ-Mex to examine the relationships between the full range of maternal and child appetitive traits. We hypothesised that the associations between mothers and their child’s corresponding appetitive traits would be positive for both food approach and food avoidance appetitive traits. As a secondary aim, we examined cross-sectional associations between appetitive traits and BMI for both mothers and children.

## Materials and methods

### Participants and procedures

Participants were recruited in the outpatient clinic of the Pediatric Department of the Hospital Civil de Guadalajara “Dr. Juan I. Menchaca” from March 2018 to February 2019. Participants were there for minor ailments or accompanying a minor relative/sibling. Those with overweight had not been previously assessed or given any treatment. Participants were recruited by PCC, through a non-probabilistic sampling technique was used, applying the following inclusion criteria: 1) mothers older than 18 years of age; 2) literate; 3) neither pregnant nor affected by any systemic illnesses, and not taking any medications that would alter appetite; 4) had a biological child aged between three and 13 years of age. Confirmation of biological kinship and interest in taking part in the study was collected. After written informed consent was obtained from the mothers—who also consented on behalf of their children after they assented to participate in the study—mothers were asked to complete the AEBQ-Esp and the CEBQ-Mex. Ethical approval was provided by the hospital’s ethics committee (No. 0222/18 HC-JIM/2018).

### Sociodemographic data

Sociodemographic characteristics were collected at baseline, which included: maternal age; education (Basic education, High school/Technical diploma, University/Postgraduate degree); occupation (housewife/unemployed, employed); marital status (married/cohabitation, divorced/separated/single); family type (nuclear or other configurations such as mono-parental, extended and joint families); and family monthly income (≤$4,500.00 MXN, >$4,500.00 MXN, >$9,000.00 MXN) registered in Mexican pesos (MXN). The child’s age at data collection was calculated from their date of birth. Mothers were asked if someone other than the parents looked after the child, and this was recorded as caregiver different from parents.

### Anthropometry

Weights and heights of the mothers and children were measured using standardized instruments (Tanita scale and Dry stadiometer) [17]. Height was recorded in centimetres (cm) and weight in kilograms (kg), to the nearest .1 kg, and subsequently used to calculate Body Mass Index (BMI) (kg/m^2^). BMI was used to categorise the mothers’ weight statuses (Healthy weight (<24.9), Overweight (25–29.9) and Obesity (<30)) and child BMI z-score (BMIz) was used to classify children’s weight statuses in accordance with WHO standards (Underweight: >-1 SD; Healthy weight: 3 to 12 years: -1 to 1 SD; Overweight 3 to 5 years: >1 SD to 3 SD; Obesity 3 to 5 years: >3 SD; Overweight 6 to 12 years: >1 SD to 2 SD; Obesity 6 to 12 years: >2 SD) [18, 19]. A total of 10 participants (1.2% of the sample) were classified with underweight and were included in the healthy weight sample due to the low number.

### Appetitive traits

Mother´s appetitive traits were measured using the AEBQ-Esp [8]. The AEBQ-Esp is a self-report questionnaire of 30 items that captures seven appetitive trait subscales. It measures three of the four CEBQ food approach subscales: Food Responsiveness (4 items; e.g. ‘I am always thinking about food’), Emotional Over-eating (5 items; e.g. ‘I eat more when I’m upset’) and Enjoyment of Food (3 items; e.g. ‘I love food’); however, the Desire to Drink scale has been removed. It also measures the same four ‘food avoidance’ subscales as are found in the CEBQ: Satiety Responsiveness (4 items; e.g. ‘I get full up easily’), Emotional Under-eating (5 items; e.g. ‘I eat less when I’m worried’), Food Fussiness (5 items; e.g. ‘I refuse new foods at first’), and Slowness in Eating (4 items; i.e. ‘I eat slowly’). Items are answered on a 5-point attitudinal Likert scale (1 = ‘strongly disagree’, 2 = ‘disagree’, 3 = ‘neither agree nor disagree’, 4 = ‘agree’, 5 = ‘strongly disagree’), which varies from the CEBQ 5-point frequency Likert scale (1 = ‘never’, 2 = ‘rarely, 3 = ‘sometimes, 4 = ‘often, 5 = ‘always’).

To measure appetitive traits in children, the CEBQ-Mex was newly developed for this study. This was a version of the CEBQ translated into Spanish and additionally cross-checked against previous non-validated Spanish versions of the CEBQ previously used in Spanish-speaking and Mexican populations [20, 21].

Prior to study commencement, to assess the validity of the CEBQ-Mex translation, structured cognitive interviews were used with a focus group of 10 mothers, using a qualitative research technique known as the “Think Aloud” method [22], previously carried out for the AEBQ-Esp validation (8). These interviews were conducted with an independent sample of mothers of different ages (21–50 years old, mean 31.1 ± 9.1 years) from the same university. These mothers were asked to read the questions aloud and to comment on their understanding of the question, as well as their mental reasoning process underlying each response. All the interviews were recorded using a digital audio recorder and transcribed to analyse responses.

The CEBQ-Mex consists of 35 items, that were answered by the mother on a 5-point Likert frequency scale (1 = ‘never’, 2 = ‘rarely, 3 = ‘sometimes, 4 = ‘often, 5 = ‘always’) (2). The CEBQ measures four ‘food approach’ subscales: Food Responsiveness (5 items; e.g. ‘My child is always asking for food’), Emotional Over-eating (4 items; e.g. ‘My child eats more when annoyed’), Enjoyment of Food (4 items; e.g. ‘My child loves food’) and Desire to Drink (3 items; e.g. ‘My child is always asking for a drink’). It also measures four ‘food avoidance’ subscales: Satiety Responsiveness (5 items; e.g. ‘My child gets full up easily’), Emotional Under-eating (4 items; e.g. ‘My child eats less when angry’), Food Fussiness (6 items; e.g. ‘My child refuses new foods at first’), and Slowness in Eating (4 items; e.g. ‘My child eats slowly’). For a complete translation of the CEBQ-Mex see S1 Table.

The mean for each AEBQ-Esp and CEBQ-Mex subscales were obtained by calculating the average score of the items corresponding to each subscale. The researcher (PCC) oversaw the data collection process to ensure all the questionnaire items were completed, which ensured no missing data, still allowing for participant privacy to answer the questionnaires.

### Statistical analysis

#### Missing data and descriptive statistics

Data were checked for missing values and outliers for height, weight or BMI, and each item’s skewness and kurtosis values were examined. The initial dataset included 855 complete mother-child dyads. A small number of dyads (n = 13; 1.5%) were removed due to incomplete maternal or child weight or height data or missing date of birth for the child. No sociodemographic differences (age, education, occupation, marital status, family type, monthly family income) or BMI differences were found between those with missing data and the final sample included in the analyses (p = .129 to p = .802). Means for each of the appetitive traits were calculated for mothers and children and checked for normality of distributions. Descriptive characteristics were derived from frequency tables and cross-tabulation. All statistical analyses were carried out in SPSS version 24.0 [23].

#### Reliability of the CEBQ-Mex

Cronbach’s alpha was used to test internal consistency or reliability for each appetitive trait for the CEBQ-Mex. Cronbach alpha values of > .70 were deemed reliable, although have been considered realistic with values of .60 [24, 25]. Omega coefficients were calculated to eliminate potential errors in the estimation of reliability [26] using JASP version 12.2 [27]. Omega coefficients > .70 were also considered reliable [26]. We also examined the reliability of the AEBQ-Esp.

#### Confirmatory factor analysis of the CEBQ-Mex

Confirmatory Factor Analysis (CFA) was carried out to examine the construct validity of the CEBQ-Mex, using JASP version 0.12.2 [27]. Goodness of fit was assessed using: the comparative fit index (CFI), the normed fit index (NFI), the Bollen’s Incremental Fit Index (IFI) and the root mean square error of approximation (RMSEA). CFI, NFI and IFI values close to .90 and RMSEA values greater than .1 are considered a poor fit [28, 29].

#### Intergenerational relationships between mothers’ and children’s appetitive traits

Pearson’s correlations were used to examine within-person associations between the scales (e.g. the associations among the children’s scales), and to assess the intergenerational relationships between mothers’ and children’s appetitive traits, as these were single measurement occasions for the AEBQ-Esp and CEBQ-Mex. This indicates the strength of the relationship for the explanation of one measure from the value of the other [30]. The alpha level was set at .05.

#### Associations between appetitive traits and BMI for mothers and children

As a secondary aim of the study, we also examined the cross-sectional associations of appetitive trait scores with BMI, for both mothers and children. Linear regressions were conducted to examine the independent associations of each appetitive trait with BMI in mothers, and each appetitive trait with BMIz in children. Possible covariates were selected by using Pearson’s correlations, t-Student test and ANOVAs to identify sociodemographic variables that were significantly associated with appetitive traits and BMI (Data available upon request). Maternal models were adjusted for maternal age, education and family type. Child models were adjusted for age, sex, maternal age and education, caregiver different from parents, family type and monthly family income.

## Results

### Descriptives

Complete data were available from 842 mother-child dyads. Maternal average age was 34.8±6.9 years old (range: 18–55 years) and the average BMI was 29.7±6.1 kg/m^2^. Nearly half of the mothers (43.7% (n = 368) were classified as living with obesity. Maternal education level was most frequently reported as ‘basic’ (61.2%, n = 515), and the majority of mothers reported being housewives (69.7%, n = 587). On average, monthly family income was low at ≤$4,500 (48.5%, n = 408). Approximately half of the children were male (53%; n = 443). The average child’s age was 8.5±2.5 years (range: 3–12.9 years), and the average BMIz was 1.5±1.6. 60.3% (n = 508) of the children had a healthy weight. A quarter of the children (n = 222; 26.4%) were taken care of by someone other than their parents (caregiver different from parents) (Table 1).

**Table 1:** Characteristics of mother-child dyads (n = 842).

Characteristics	Mean ± SD	n (%)
**Mothers (n = 842)**		
Age (years)	34.76± 6.9	
BMI (kg/m^2^)	29.68± 6.1	
Healthy weight		187 (22.2)
Overweight		287 (34.1)
Obese		368 (43.7)
Education		
Basic education		515 (61.2)
High school/Technical diploma		252 (29.9)
University/Postgraduate degree		75 (8.9)
Occupation		
Housewife/Unemployed		587 (69.7)
Employed		255 (30.3)
Marital status		
Married/cohabitation		640 (76.0)
Divorced/Separated/Single		202 (24.0)
Family type		
Nuclear		551 (65.4)
Other^1^		291 (34.6)
Monthly family income^2^		
<$4,500		408 (48.5)
≥$4,500 and <$9,000		274 (32.5)
≥$9,000		160 (19.0)
**Children (n = 842)**		
Age (years)	8.51 ± 2.2	
Sex		
Male		443 (52.6)
Female		399 (47.4)
Weight (kg)	35.78 ± 14.5	
Height (cm)	129.95 ± 15.7	
BMI-z^3^	1.47 ± 1.6	
Healthy weight		508 (60.3)
Overweight		227 (27.0)
Obese		107 (12.7)
Caregiver different from parents^4^		
No		620 (73.6)
Yes		222 (26.4)

^1^Monoparental, extended, joint.

^2^Mexican pesos. At the time of paper submission (August, 2021), $4,500 Mexican pesos equals $225 U.S. dollars and $9,000 Mexican pesos equals $450 U.S. dollars per month.

^3^WHO. Healthy weight: 3 to 12 years: -1 to 1 SD; Overweight 3 to 5 years: >1 SD to 3 SD (risk of overweight and age were included in this category for statistical analysis); Overweight 6 to 12 years: >1 SD a 2 SD; Obesity 3 to 5 years: >3 SD; Obesity 6 to 12 years: >2 SD.

^4^Mothers were asked if someone other than the parents looked after the child.

### CEBQ translation and “Think Aloud”

An independent sample of mothers were invited to say ‘out loud’ what they thought about each CEBQ-Mex item. Ten mothers who provided informed consent, took part. These mothers were aged 21 to 50 years (31.1 ± 9.1 years old), six were housewives and the rest employed, and all reported primary school education (≥6 years). No items had to be changed in the questionnaires as mothers understood all questions as intended. The children’s ages ranged from four to 11 years (6.9 ± 2.8 years), and 50% (n = 5) were male.

### Reliability of the CEBQ-Mex and AEBQ-Esp

Means and standard deviations for the CEBQ-Mex and the AEBQ-Esp appetitive trait subscale scores are shown in Table 2. Most of the AEBQ-Esp scales showed good reliability [Cronbach’s alphas (.60–0.83); Omega (.62–0.83)]; however, Food Fussiness and Satiety Responsiveness had only moderate omega values of .62 and .65, respectively. The CEBQ-Mex also was found to be a reliable instrument [Cronbach’s alphas (.68-.86); Omega (.71-.87)] (Table 2).

**Table 2:** Descriptive statistics (mean ± standard deviation) and internal consistency estimates (Cronbach’s α and Omega ω) for the AEBQ-Esp (n = 842) and CEBQ-Mex (n = 842).

Appetitive Traits	AEBQ-Esp				CEBQ-Mex			
	Mean ± SD	Cronbach’s α (and Omega ω)	Omega ω 95.0% Confidence Interval		Mean ± SD	Cronbach’s α (and Omega ω)	Omega ω 95.0% Confidence Interval	
			Lower	Upper			Lower	Upper
**Food Approach subscales**								
Food responsiveness	2.57 ± .77	.66 (.69)	.62	.70	2.71 ± 1.12	.86 (.87)	.85	.88
Emotional over-eating	2.43 ± .95	.83 (.83)	.81	.85	2.17 ± 0.94	.78 (.79)	.76	.81
Enjoyment of food	3.83 ± .76	.71 (.73)	.67	.74	4.03 ± 0.84	.80 (.81)	.78	.82
Desire to drink	–	–	–	–	3.45 ± 1.16	.84 (.84)	.82	.85
**Food Avoidance subscales**								
Satiety responsiveness	2.68 ± .82	.64 (.65)	.60	.68	2.53 ± 0.85	.74 (.75)	.71	.77
Emotional under-eating	2.77 ± .96	.80 (.80)	.78	.82	2.62 ± 0.93	.68 (.71)	.64	.71
Food fussiness	2.43 ± .70	.60 (.62)	.53	.62	2.83 ± 0.84	.77 (.78)	.75	.80
Slowness in eating	2.87 ± .88	.68 (.70)	.64	.71	2.56 ± 0.94	.72 (.74)	.69	.75

^1 1^Cronbach’s α threshold for acceptable values: 0.7–0.90 (24,25).

^2^Omega ω threshold for acceptable values: 0.65–0.90 (26).

### Confirmatory factor analysis of the CEBQ-Mex

CFA of the original factor structure of the CEBQ revealed a valid eight subscales instrument with an acceptable model fit [RMSEA = .065; CFI = .840, NFI = .805; IFI = .842; and χ^2^(df = 532) = 2939.51, p < .001] [28, 29] (Table 3).

**Table 3:** Confirmatory factor analysis and fit indices of the CEBQ-Mex.

Model fit indices	*n* = 842
X^2^ (degrees of freedom)	2939.51 (532)
X^2^ *p* value	< .001
RMSEA	.065
CFI	.840
NFI	.805
IFI	.842

Comparative fit index (CFI); Bollen’s Incremental Fit Index (IFI); Normed fit index (NFI); Root mean square error of approximation (RMSEA).

Factor loadings for all CEBQ-Mex factors are shown in Table 4.

**Table 4:** Factor loadings for CEBQ-Mex items estimated from principal component analysis (n = 842).

	Items	Standardized factor loadings CFA– 8 factors Total sample (*n* = 842)
		λ (95% CI)
FR	CEBQ19	1.13 (1.04, 1.21)
	CEBQ23	.83 (.74, .93)
	CEBQ25	1.07 (.98, 1.15)
	CEBQ30	1.07 (.99, 1.15)
	CEBQ31	1.13 (1.04, 1.21)
EOE	CEBQ5	.67 (.60, .75)
	CEBQ8	.77 (.70, .84)
	CEBQ11	.97 (.88, 1.06)
	CEBQ18	.96 (.89, 1.04)
EF	CEBQ1	.67 (.62, .73)
	CEBQ3	.68 (.63, .74)
	CEBQ4	.81 (.74, .89)
	CEBQ16	.84 (.77, .92)
DD	CEBQ10	1.06 (.98, 1.14)
	CEBQ24	1.16 (1.09, 1.24)
	CEBQ34	.95 (.87, 1.04)
SR	CEBQ12	.80 (.72, .88)
	CEBQ26	.81 (.74, .89)
	CEBQ33	.66 (.57, .74)
	CEBQ35	.74 (.66, .82)
	CEBQ14*	.66 (.58, .75)
EUE	CEBQ6	.58 (.48, .69)
	CEBQ9	.49 (.41, .57)
	CEBQ20	1.02 (.94, 1.10)
	CEBQ22	1.02 (.93, 1.10)
FF	CEBQ2	.63 (.55, .71)
	CEBQ7	.83 (.75, .92)
	CEBQ13*	.72 (.63, .81)
	CEBQ17	.86 (.78, .94)
	CEBQ21*	.78 (.71, .86)
	CEBQ27*	.60 (.52, .68)
SE	CEBQ28	.93 (.84, 1.01)
	CEBQ29	.86 (.78, .94)
	CEBQ32	.86 (.77, .95)
	CEBQ15*	.56 (.47, .66)

FR = Food Responsiveness; EOE = Emotional Over-Eating; EF = Enjoyment of Food; DD = Desire to Drink; SR = Satiety Responsiveness; EUE = Emotional Under-Eating; FF = Food Fussiness; SE = Slowness in Eating; CEBQ-Mex = Child Eating Behaviour Questionnaire Mexican Spanish Version.

### Intergenerational relationships between mothers’ and children’s appetitive traits

#### Associations between maternal and child scales

To show the relationships between maternal and child appetitive traits, correlations between concordant subscales were examined. As hypothesised, maternal appetitive traits were significantly and positively correlated with their child´s equivalent appetitive traits, except for Slowness in Eating (r = .01; p = .820) (Table 5). Correlations were of small to moderate magnitude [Food Responsiveness (r = .24; p < .001), Emotional Over-Eating (r = .29; p < .001), Enjoyment of Food (r = .16; p = .009), Satiety Responsiveness (r = .16; p < .001), Emotional Under-Eating (r = .34; p < .001) and Food Fussiness (r = .14; p < .001)].

**Table 5:** Pearson’s correlations between the AEBQ-Esp subscales (n = 842 mothers) and the CEBQ-Mex subscales (n = 842 children).

CEBQ-Mex subscales	AEBQ-Esp subscales						
	FR	EOE	EF	SR	EUE	FF	SE
Food Approach subscales							
Food responsiveness	**.24** ^**1**^	.17^**1**^	.06	.14^**1**^	.14^**1**^	.04	.05
Emotional over-eating	.22^**1**^	**.29** ^**1**^	0	.19^**1**^	.20^**1**^	.13^**1**^	.09^2^
Enjoyment of food	.06	.05	**.16** ^**1**^	.02	.01	-.11^**1**^	.05
Food Avoidance subscales							
Satiety responsiveness	.10^2^	.05	.02	**.16** ^**1**^	.08^2^	.03	-.02
Emotional under-eating	.25^**1**^	.27^**1**^	.07^2^	.19^**1**^	**.34** ^**1**^	.09	.04
Food fussiness	.09^**1**^	.06	-.02	.07^2^	.11^**1**^	**.14** ^**1**^	0
Slowness in eating	.07^2^	.03	-.03	.09^2^	.13^**1**^	.01	.01

FR = Food Responsiveness; EOE = Emotional Over-Eating; EF = Enjoyment of Food; SR = Satiety Responsiveness; EUE = Emotional Under-Eating; FF = Food Fussiness; SE = Slowness in Eating; AEBQ-Esp = Adult Eating Behaviour Questionnaire Spanish version; CEBQ-Mex = Child Eating Behaviour Questionnaire Mexican Spanish version.

^1^Correlation is significant at the 0.01 level (2-tailed).

^2^Correlation is significant at the 0.05 level (2-tailed).

Associations among the maternal scales and among the children’s scales are shown in S2 Table.

### Associations between appetitive traits and BMI for mothers and children

In S3 Table we include the results for the non-adjusted and adjusted linear regression analyses of maternal appetitive traits with BMI. In mothers, we found a significant positive association of Emotional Over-Eating with BMI [β = .11 (.23, 1.09)] (p = .006). In contrast, Enjoyment of Food was negatively associated with BMI [β = -.07 (-1.20, -.12 (p = .047). In children, Food Responsiveness [β = .10 (.02, .31) (p < .029)], Emotional Over-Eating [β = .18 (.15, .42) (p < .000)] and Food Fussiness [β = .08 (.00, .25) (p = .049)] were significantly positively associated BMI-z scores. On the other hand, Satiety Responsiveness [β = -.15 (-.34, -.09) (p = .001)], Emotional Under-Eating [β = -.10 (-.27, -.03) (p = .01)] and Slowness in Eating [β = -.11 (-.29, -.06) (p = .003)] were significantly and negatively associated with BMI-z scores in children.

## Discussion

The CEBQ-Mex is the first translation of the CEBQ into Mexican Spanish, which has been found to be reliable and valid, and replicates the original 8-factor structure of the CEBQ. As hypothesised, most maternal appetitive traits measured using the AEBQ-Esp were positively and significantly associated with their child’s counterpart, measured using the CEBQ-Mex, which provide evidence for intergenerational transmission of appetite between mothers and their children, observed in the significant correlations between six appetitive traits among mother-child dyads. Slowness in Eating was the only trait that was not significantly correlated between mothers and children.

The CEBQ-Mex was found to be reliable, with a robust factor structure comparable to the original version (RMSEA .065) [31]. The CEBQ has previously been translated into Mexican Spanish and administered to 640 adult caregivers of 6–12-year-old children. However, only six subscales were identified through PCA, and confirmatory factor analysis was not carried out [32]. It has also previously been used to report children’s appetitive traits in the same city where we recruited our participants [20, 21]. Results were similar to the Chilean validation of the CEBQ in 126 obese, 44 overweight and 124 normal-weight Chilean children between the ages of 6 to 2 years-old where PCA was carried out in both sexes and the 8-factor structure was confirmed [33].

Maternal food approach appetitive traits were positively associated with the corresponding subscales of the child, except for Slowness in Eating. Miller and colleagues (2020) also reported positive correlations between mothers and their children for Emotional-Over Eating and Food Responsiveness (r = 0.29 and r = 0.21, respectively), which were of a similar magnitude to those observed in this study [14]. The main aim of Miller and colleagues’ (2020) research was to establish if parental feeding practices mediated the associations between maternal and child Emotional Over-Eating and Food Responsiveness specifically, so only these two subscales were examined between mothers and their children [14]. We have been able to show that all the appetitive traits, except for Slowness in Eating, were consistently positively correlated between mothers and their children. The null association shown between the Slowness in Eating scale between mothers and children warrants further research.

Parent-child correlations for appetitive traits may reflect both genetic inheritance and shared environmental influences (such as parental modelling of eating behaviours to children, or parental feeding practices). Twin studies have established that most appetitive traits are under moderate genetic influence [34, 35], and the Behavioural Susceptibility Theory (BST) hypothesises that they characterise some of the neuro-behavioural mechanisms that underpin and mediate genetic susceptibility to obesity (BST) [36]. BST proposes that individuals who inherit tendencies towards food approach traits are predisposed towards overeating when exposed to a food permissive environment. There are also important environmental shapers of appetitive traits, and twin studies indicate that these are especially prominent for emotional under- and overeating.

In this study, the smallest correlations were observed for Food Fussiness (r = .14), Enjoyment of Food and (r = .16) and Satiety Responsiveness (r = .16), while the highest correlations were those observed for Emotional Over-Eating (r = .29) and Emotional Under-Eating (r = .34). The higher correlations for emotional eating might reflect the strong shared environmental influences on emotional eating reported in twin studies of British children [37]. These findings highlight that emotional eating tends to run strongly in families, with twin siblings, and parents and children strongly resembling one another for these eating behaviours. On the other hand the low mother-child correlation for Food Fussiness was somewhat surprising given its strong genetic influence reported in twin studies of children [38–41]. However, shared environmental influences are low, and genetic influences on Food Fussiness may decline as children mature into adulthood, as is observed for most behavioural traits, with the non-shared environment becoming the more dominant source of variation at older ages [38]. This change in the relative influence of genetic and unique environmental factors would be reflected in a lower mother-child correlation for this trait because the genetic factors at play for mothers and their children may be different in type or magnitude, and there is little if any shared environmental influence on this trait. Food responsiveness (r = .24) and satiety responsiveness (r = .16) had small to moderate associations, which were somewhat surprising given the high heritability estimates for these traits observed in a large sample of British twin children (75% and 63% respectively) [42]. This may reflect different types or magnitudes of genetic influences on these traits in children and adults. Importantly, estimates of genetic and environmental influence can vary considerably with age and sample. Nevertheless, these results indicate strong intergenerational transmission of a number of weight-related appetitive traits, highlighting the importance of promoting healthy eating practices in for both parents and their children, taking a family-wide intervention approach [43].

The secondary aim of this study was to examine associations of appetitive traits and BMI among mothers and children. We found a significant positive association between Emotional Over-Eating and BMI in mothers. These results are similar to other AEBQ validation studies [8, 10]. However, Enjoyment of Food was negatively associated with BMI in mothers. This outcome contrasts with previous AEBQ validation studies. This finding could suggest social desirability bias insofar as women struggling with their weight may feel ashamed to admit that they like food and enjoy eating. This may be even more pronounced in a country in which overweight and obesity rates in women are at 76.8%, and widely proclaimed to be a public health crisis [44]. It may also be the case that women who are struggling with their weight no longer enjoy food or eating, due to body image dissatisfaction and disordered eating cognitions.

Among children, Food Responsiveness and Emotional Over-Eating were significantly positively associated with BMI-z scores. Satiety Responsiveness, Emotional Under-Eating and Slowness in Eating were also significantly negatively associated with BMI-z scores. These results are comparable to those found in a recent systematic review and meta-analysis of 67 CEBQ studies [6]. In this sample of children, Food Fussiness was positively significantly associated with BMI-z scores, in line with data collected in children with overweight and obesity in the same hospital that this sample were recruited from [21]. Although these results may seem surprising, fussier children are often offered foods that are well liked by all children, which tend to be foods higher in energy density [21].

This is the first study to provide evidence for intergenerational transmission of appetite between mothers and their children, reflected in significant correlations between six appetitive traits among mother-child dyads, which capture both genetic and shared environmental influences. The CEBQ-Mex offers a much-needed psychometric instrument for the comprehensive measurement of appetitive traits among Mexican children. It was found to be reliable, with an acceptable factor structure that compares with the original version. However, as a parent report measure, reporting bias by the mother cannot be ruled out. For example, a mother’s perceptions of her child’s weight may influence how she describes her child’s eating behaviours, and, in this sample, a large percentage of children had overweight or obesity (close to 40%). Also, approximately a quarter of the caregivers in the sample were not the mothers of the children, which could potentially not accurately report on the child’s appetite traits, due to insufficient interaction with the child. The AEBQ-Esp had lower than ideal reliability estimates for Food Fussiness (ω = .62) and Satiety Responsiveness (ω = .69). However, even if Omega values are slightly lower than .70, they are still considered acceptable [26]. Nevertheless, it is important to consider that in general the mothers of the children who attended the hospital in the present study had low educational attainment [45], which may have impacted their level of understanding of the questionnaire. In this respect, the study had limited generalisability. Future studies should replicate these findings in a more socioeconomically diverse sample.

Future longitudinal research should explore the bidirectional nature of the relationship between maternal and child appetitive traits, especially in relation to the risk of overweight and obesity, as well as other contributing factors such as parental feeding practices, food preferences and food choices. More research is also needed to develop interventions that help parents recognise strongly expressed appetitive traits in themselves and their children, and to understand how these affect their own food choices, modelling of eating behaviour, and feeding practices [14].

## Conclusions

Mothers and their children’s appetitive traits tend to be positively correlated, such that children’s eating styles reflect, to some extent, those of their mothers. Future studies should seek to understand better how parental appetitive traits are passed on from parent to child. This is important, because for both mothers and their children, food approach and food avoidance traits are associated with BMI, and this may provide healthcare professionals and public health programs with an opportunity to optimise energy balance–related eating behaviours within a family unit.

## Supporting information

S1 Table Full set of CEBQ and CEBQ-Mex items. (DOCX)

S2 Table Within-person Pearson’s correlations between the AEBQ-Esp (n = 842 mothers) and CEBQ-Mex (n = 842 children) subscales. (DOCX)

S3 Table Linear regressions to examine the independent associations between each AEBQ-Esp appetitive trait and BMI (n = 842 mothers) and each CEBQ-Mex appetitive trait and BMIz (n = 842 children). (DOCX)
