# Supplementary material for: Intergenerational transmission of appetite: Associations between mother-child dyads in a Mexican population
Source: PLoS One. 2022 Mar 15;17(3):e0264493. doi: 10.1371/journal.pone.0264493 (PMC8923510; doi:10.1371/journal.pone.0264493)
Supplement: S1 Table — (DOCX) [file pone.0264493.s001.docx]

| **CEBQ^1^** | **Subescales** | | **CEBQ-Mex^2^** |
| --- | --- | --- | --- |
| **Food Aproach subscales; Sub-escalas pro-ingestión** | | | |
| Given the choice, my child would eat most of the time | Food Responsiveness | Respuesta a los Alimentos | Si mi hijo pudiera escoger, se la pasaría comiendo la mayor parte del tiempo |
| Even if my child is full up s/he finds room to eat his/her favourite food |  |  | Aún cuando mi hijo(a) se siente lleno encuentra el espacio para comer su alimento favorito |
| If given the chance, my child would always have food in his/her mouth |  |  | Si se le diera la chance, mi hijo(a) siempre tendría un alimento en su boca |
| My child is always asking for food |  |  | Mi hijo(a) siempre está pidiendo comida |
| If allowed to, my child would eat too much |  |  | Si se le permitiera, mi hijo(a) comería demasiado |
| My child eats more when annoyed | Emotional over-eating | Sobre-  Alimentación Emocional | Mi hijo(a) come más cuando esta irritado(a) o enfadado(a) |
| My child eats more when worried |  |  | Mi hijo(a) come más cuando esta preocupado(a) |
| My child eats more when anxious |  |  | Mi hijo(a) come más cuando esta ansioso(a) |
| My child eats more when s/he has nothing else to do |  |  | Mi hijo(a) come más cuando no tiene nada que hacer |
| My child loves food | Enjoyment of Food | Disfrute de los Alimentos | A mi hijo(a) le toma más de 30 minutos terminarse sus alimentos |
| My child enjoys eating |  |  | Mi hijo(a) come cada vez más lento durante el transcurso de una comida |
| My child looks forward to mealtimes |  |  | Mi hijo(a) come despacio |
| My child is interested in food |  |  | Mi hijo(a) termina sus alimentos rápidamente |
| **Food Avoidance subescales; Sub-escalas anti-ingestión** | | | |
| My child leaves food on his/her plate at the end of a meal | Satiety Responsiveness | Respuesta a la Saciedad | Mi hijo(a) deja alimentos en su plato al final de una comida |
| My child gets full before his/her meal is finished |  |  | Mi hijo(a) se siento lleno(a) antes de que termine su comida |
| My child cannot eat a meal if s/he has had a snack just before |  |  | Mi hijo(a) no puede comerse su comida si ha comido un bocadillo justo antes |
| My child gets full up easily |  |  | Mi hijo(a) se siente lleno(a)/ satisfecho(a), muy fácilmente |
| My child has a big appetite |  |  | Mi hijo(a) tiene un gran apetito |
| My child eats less when angry | Emotional Under-Eating | Sub-  Alimentación Emocional | Mi hijo(a) come menos cuando esta enojado(a) |
| My child eats less when upset |  |  | Mi hijo(a) come menos cuando esta molesto(a) |
| My child eats more when she is happy |  |  | Mi hijo(a) come más cuando está contento(a) |
| My child eats less when s/he is tired |  |  | Mi hijo(a) come menos cuando está cansado(a) |
| My child decides that s/he doesn’t like a food, even without tasting it | Food Fussiness | Actitud remilgosa | Mi hijo(a) decide que no le gusta un alimento, antes de probarlo |
| My child refuses new foods at first |  |  | Al principio mi hijo(a) rechaza nuevos alimentos |
| My child is difficult to please with meals |  |  | Mi hijo(a) es difícil de complacer con alimentos |
| My child enjoys tasting new foods |  |  | A mi hijo(a) le gusta probar alimentos nuevos |
| My child enjoys a wide variety of foods |  |  | Mi hijo(a) disfruta una gran variedad de alimentos |
| My child is interested in tasting foods s/he hasn´t tasted before |  |  | A mi hijo(a) le interesa probar alimentos nuevos que no ha probado antes |
| My child takes more than 30 minutes to finish a meal | Slowness in Eating | Lentitud para comer | A mi hijo(a) le toma más de 30 minutos terminarse sus alimentos |
| My child eats more and more slowly during the course of a meal |  |  | Mi hijo(a) come cada vez más lento durante el transcurso de una comida |
| My child eats slowly |  |  | Mi hijo(a) come despacio |
| My child finishes his/her meal quickly |  |  | Mi hijo(a) termina sus alimentos rápidamente |

**Supporting** **information 1.** Full set of CEBQ and CEBQ-Mex items

^1^Response option: Strongly disagree, Disagree, Neither agree nor disagree, Agree, Strongly agree.

^2^Opción de respuestas: Muy en desacuerdo, En desacuerdo, Ni en acuerdo ni en desacuerdo, En acuerdo, Muy en acuerdo

# Intergenerational transmission of appetite: Associations between mother-child dyads in a Mexican population.

**Hunot-Alexander C^1†^, Curiel CP^1†^, Romero-Velarde E^1^, Vásquez-Garibay EM^1^, Mariscal A^1^, Casillas E^2^, Smith A^3*^, Llewellyn, C^3‡^.**

^1^ Instituto de Nutrición Humana, CUCS, Universidad de Guadalajara.

^2^ Hospital Civil “Dr Juan I Menchaca”, Guadalajara, Jalisco.

^3^ Department of Behavioural Science and Health, University College London.

* Corresponding author: [andrea.smith@ucl.ac.uk](mailto:andrea.smith@ucl.ac.uk)
