# Supplementary material for: Intergenerational transmission of appetite: Associations between mother-child dyads in a Mexican population
Source: PLoS One. 2022 Mar 15;17(3):e0264493. doi: 10.1371/journal.pone.0264493 (PMC8923510; doi:10.1371/journal.pone.0264493)
Supplement: S2 Table — (DOCX) [file pone.0264493.s002.docx]

**S2. Table.** Within-person Pearson’s correlations between the AEBQ-Esp (n=842 mothers) and CEBQ-Mex (n=842 children) subscales

Intergenerational transmission of appetite: Associations between mother-child dyads in a Mexican population. **Hunot-Alexander C^1†^, Curiel-Curiel CP^1†^, Romero-Velarde E^1^, Vásquez-Garibay EM^1^, Mariscal A^1^, Casillas E^2^, Smith A^3*^, Llewellyn, C^3‡^.**

^1^ Instituto de Nutrición Humana, CUCS, Universidad de Guadalajara.

^2^ Hospital Civil “Dr Juan I Menchaca”, Guadalajara, Jalisco.

^3^ Department of Behavioural Science and Health, University College London.

* Corresponding author: [andrea.smith@ucl.ac.uk](mailto:andrea.smith@ucl.ac.uk)

**S2. Table.** Within-person Pearson’s correlations between the AEBQ-Esp (n=842 mothers) and CEBQ-Mex (n=842 children) subscales

| **AEBQ-Esp subscales** | EOE | EF | DD | SR | EUE | FF | SE |
| --- | --- | --- | --- | --- | --- | --- | --- |
|  |  |  |  |  |  |  |  |
| Food Approach subscales |  |  |  |  |  |  |  |
| Food responsiveness | .51^1^ | .21^1^ | – | 1 | .19^1^ | .04 | .04 |
| Emotional over-eating | 1 | .11^1^ | – | .13^1^ | .12^1^ | .05 | -.01 |
| Enjoyment of food |  | 1 | – | 0 | 1 | -.23^1^ | .02 |
| Food Avoidance subscales |  |  |  |  |  |  |  |
| Satiety responsiveness |  |  |  | 1 | .40^1^ | .17^2^ | .29^1^ |
| Emotional under-eating |  |  |  |  | 1 | .04 | .18^1^ |
| Food fussiness |  |  |  |  |  | 1 | .09^1^ |
| Slowness in eating |  |  |  |  |  |  | 1 |
| **CEBQ-Mex subscales** | EOE | EF | DD | SR | EUE | FF | SE |
|  |  |  |  |  |  |  |  |
| Food Approach subscales |  |  |  |  |  |  |  |
| Food responsiveness | .61^1^ | .42^1^ | .47^1^ | -.22^1^ | .26^1^ | -.04 | -.20^1^ |
| Emotional over-eating | 1 | .28^1^ | .32^1^ | -.06 | .43^1^ | .12^1^ | -.11^2^ |
| Enjoyment of food |  | 1 | .14^1^ | -.53^1^ | .04 | -.46^1^ | -.34^1^ |
| Desire to drink |  |  | 1 | .07 | .32^1^ | .08^2^ | .037 |
| Food Avoidance subscales |  |  |  |  |  |  |  |
| Satiety responsiveness |  |  |  | 1 | .27^1^ | .43^1^ | .05^1^ |
| Emotional under-eating |  |  |  |  | 1 | .19^1^ | .16^1^ |
| Food fussiness |  |  |  |  |  | 1 | .18^1^ |
| Slowness in eating |  |  |  |  |  |  | 1 |

EOE= Emotional Over-Eating; EF= Enjoyment of Food; SR= Satiety Responsiveness; EUE=Emotional Under-Eating; FF= Food Fussiness; SE= Slowness in Eating; AEBQ-Esp=Adult Eating Behaviour Questionnaire Spanish Version; CEBQ-Mex=Child Eating Behaviour Questionnaire Mexican Spanish version.

^1^Correlation is significant at the 0.01 level (2-tailed).

^2^Correlation is significant at the 0.05 level (2-tailed).
