# Supplementary material for: Intergenerational transmission of appetite: Associations between mother-child dyads in a Mexican population
Source: PLoS One. 2022 Mar 15;17(3):e0264493. doi: 10.1371/journal.pone.0264493 (PMC8923510; doi:10.1371/journal.pone.0264493)
Supplement: S3 Table — (DOCX) [file pone.0264493.s003.docx]

Supporting information 3. **Pearson’s correlations, t-Student test or ANOVA between appetitive traits and sociodemographic variables of the mothers and children**

Intergenerational transmission of appetite: Associations between mother-child dyads in a Mexican population. **Hunot-Alexander C^1†^, Curiel-Curiel CP^1†^, Romero-Velarde E^1^, Vásquez-Garibay EM^1^, Mariscal A^1^, Casillas E^2^, Smith A^3*^, Llewellyn, C^3‡^.**

^1^ Instituto de Nutrición Humana, CUCS, Universidad de Guadalajara.

^2^ Hospital Civil “Dr Juan I Menchaca”, Guadalajara, Jalisco.

^3^ Department of Behavioural Science and Health, University College London.

* Corresponding author: [andrea.smith@ucl.ac.uk](mailto:andrea.smith@ucl.ac.uk)

**Table A3.1. Linear regressions* to examine the independent associations between each AEBQ-Esp appetitive trait and BMI (n=842 mothers) and each CEBQ-Mex appetitive trait and BMIz (n=842 children)**

| **AEBQ-Esp subscales** | **BMI** | |
| --- | --- | --- |
|  | **Un-adjusted (β)^4^**  **95% CI** | **Adjusted^3^ (β)^4^**  **95% CI** |
| **Food Approach subscales** |  |  |
| Food responsiveness | -.07 (-1.17, .10) | -.04 (-.73, .27) |
| Emotional over-eating | **.15^1^ (.43, 1.44)** | **.11^1^ (.19, 1.16)** |
| Enjoyment of food | **-.08^1^ (-1.25, -.11)** | **-.07^2^ (-88, -.00)** |
| **Food Avoidance subscales** |  |  |
| Satiety responsiveness | .04 (-.27, .87) | .04 (-.23, .71) |
| Emotional under-eating | -.01 (-.53, .42) | -.01 (-.50, .41) |
| Food fussiness | .02 (-.47, .77) | .02 (-.30, .57) |
| Slowness in eating | **-.07^2^ (-.99, .01)** | -.07 (-.85, .02) |
| **CEBQ-Mex subscales** | **BMIz** | |
|  | **Un-adjusted (β)^4^**  **95% CI** | **Adjusted^5^ (β)^4^**  **95% CI** |
| **Food Approach subscales** |  |  |
| Food responsiveness | .08 (-.01, .24) | **.10^2^ (.02, .31)** |
| Emotional over-eating | **.20^1^ (.19, .48)** | **.18^1^ (.15, .42)** |
| Enjoyment of food | -.01 (-.17, .15) | -.10 (-.15, .12) |
| Desire to drink | .04 (-.04, .15) | .03 (-.07, .16) |
| **Food Avoidance subscales** |  |  |
| Satiety responsiveness | **-.16^1^ (-.45, -.13)** | **-.15^1^ (-.34, -.09)** |
| Emotional under-eating | **-.10^1^ (-.31, -.05)** | **-.10^1^ (-.27, -.03)** |
| Food fussiness | **.09^2^ (.02, .31)** | **.08^2^ (.00, .25)** |
| Slowness in eating | **-.13^1^ (-.34, -.09)** | **-.11^1^ (-.29, -.06)** |

* Separate linear regressions were ran for each appetitive trait.

^1^Correlation is significant at the 0.01 level (2-tailed).

^2^Correlation is significant at the 0.05 level (2-tailed).

^3^Adjusted for mother’s education and family type.

^4^β values are unstandarised.

^5^Adjusted for child age, sex and caregiver different from parents, family type and parent’s marital status

CI: Confidence Interval
